# Squeeze Film Damping Effect on Different Microcantilever Probes in Tapping Mode Atomic Force Microscope

**DOI:** 10.1155/2020/8818542

**Published:** 2020-11-13

**Authors:** Yan Sun, Jing Liu, Kejian Wang, Zheng Wei

**Affiliations:** College of Mechanical and Electrical Engineering, Beijing University of Chemical Technology, Beijing 100029, China

## Abstract

During the operation of tapping mode atomic force microscope (TM-AFM), the gap between the cantilever and sample surface is very small (several nanometers to micrometers). Owing to the small gap distance and high vibration frequency, squeeze film force should be considered in TM-AFM. To explore the mechanism of squeeze film damping in TM-AFM, three theoretical microcantilever simplified models are discussed innovatively herein: tip probe, ball probe, and tipless probe. Experiments and simulations are performed to validate the theoretical models. It is of great significance to improve the image quality of atomic force microscope.

## 1. Introduction

Tapping mode atomic force microscope (TM-AFM) is widely used to observe samples at true atomic resolution [1, 2]. AFM phase images provide more significant information than topographic images, such as information regarding adhesion, elasticity, viscoelasticity, stiffness, and chemical composition [3, 4]. Phase images and the quality factor *Q* are directly related to energy dissipation between the tip and sample [5, 6]. Many energy dissipation mechanisms, such as adhesion hysteresis, capillary interactions, air damping, and plastic deformation, have been discussed in previous studies [7–13]. Increasing the cantilever quality factor *Q* improves imaging sensitivity and helps to provide high-resolution phase images [14, 15]. In addition, the accurate identification of each dissipation mechanism is helpful for improving the imaging accuracy.

At present, the research on squeeze film damping is mainly focused on microelectromechanical systems (MEMS) [16], and there are few studies on squeeze film damping in TM-AFM. In related experimental studies, Garcia et al. [17, 18] used tune resonance curves (frequency sweep from low to high) to investigate the change of resonance amplitude with the decrease of tip-sample distance. In their study, the amplitude was considered as constant when the tip-sample distance was large enough. However, in fact, the amplitude exhibited a slight change before the truncated appeared, which was not presented in their papers. This experiment agrees with the study of Hoummady [19], stating that these phenomena can be explained by squeeze film damping. The experiment was carried out by Yang et al. [20] with a common probe (tip probe) in the TM-AFM, which has the advantage of more accurate tip-sample distance. In related theoretical studies, many scholars solved the Reynolds equation to obtain the influence of squeeze film damping on the dynamics of the cantilever beam, but they did not consider the effect of different tip shapes on the squeeze film damping [21–24]. Lévêque et al. [25] proposed that the squeeze film damping force is related to the geometry of the tip cantilever system and a related expression can be obtained. In addition, it was found that their conclusions were in quantitative agreement with the experimental data, which clearly showed the effect of viscosity in all distances.

In this study, we demonstrate the squeeze film damping mechanism of different probes in TM-AFM and analyze the effects of squeeze film damping on amplitude and the quality factor *Q*. The results of the theoretical models are compared with those obtained via the experiment using three types of AFM probes (tip probe, ball probe, and tipless probe).

## 2. Experiment Procedure

### 2.1. Cantilever Types

**Figure 1:**
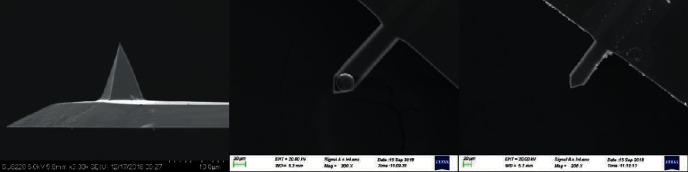
SEM images of the three different types of probes, i.e., tip probe, ball probe, and tipless probe.

To verify the squeeze film damping model, we designed an experiment to test amplitude change when the tip-sample distance was in the micron range. Tip probe (AppNano AN-NSC10-2 thickness: 4 *μ*m, length: 125 *μ*m, width: 30 *μ*m, resonance frequency: 200-400 kHz, force constant: 40 N/m, coating: Al, material: Si, tip radius: 10 nm, tip height: 15 *μ*m), ball probe (attach SiO_2_ spheres to the tipless cantilever, cantilever type: Nanosensors TL-NCH-10, particle shape: ball particle, diameter: 10 *μ*m, particle material SiO_2_), and tipless probe (Nanosensors TL-NCH-10, thickness: 4 *μ*m, length: 125 *μ*m, width: 30 *μ*m, resonance frequency: 204-479 kHz, force constant: 40 N/m, coating: none, material: Si) were used as shown in Figure 1 to analyze the effects of squeeze film damping. A Bruker Dimension Icon AFM was used in these experiments.

### 2.2. Experiment Procedure

**Figure 2:**
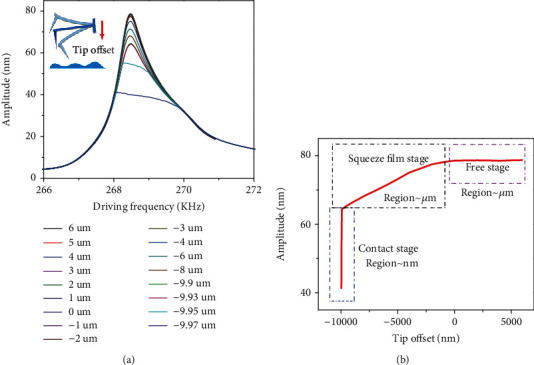
(a) Tip probe experimental sweep curves for different tip offset (relative tip-sample distances). (b) Maximum amplitude of each sweep curve.

In the TM-AFM autotune function, the tip offset was used to engage the cantilever on the sample stepwise. The engaged distance per step was controlled within a few micrometers or nanometers. Experimental resonance curves for different tip offset are shown in Figure 2(a). The dependence of the cantilever oscillation amplitude on the tip offset is shown in Figure 2(b).

In Figure 2(b), the tuning curve can be divided into three stages. When the tip-sample distance was relatively high, this area can be defined as the free stage, the amplitude of the tip kept constant. As the tip engaged on the sample, the probe gradually entered the squeeze film damping stage; in this stage, the tip offset led the amplitude to decline slowly. The last stage is the contact stage; in this stage, the tip touched the sample on one side. In the contact stage, the tip offset led to the rapid change in amplitude. This stage can be simply described using a contact model, as the tip-sample contact caused the truncation in Figure 2(b).

## 3. Experimental Results and Theoretical Calculation

### 3.1. Van der Waals Forces

It is well known that van der Waals forces should be notices when the distances between objects are small. To determine the effects of the van der Waals forces on the cantilever, force curves can be used to verify the action distance of these forces. The “jump into contact” phenomenon occurs at a tip-sample distance of several nanometers. Van der Waals forces between the tip and sample can be ignored when the tip-sample distance is of the order of microns. The squeeze film force is much larger than the van der Waals forces for micron-scale tip-sample distances [21]. Furthermore, it is necessarily pointed that the samples should be placed for a long time before the experiment to eliminate the effect of electrostatic forces.

### 3.2. Ball Probe

For the ball probe, a micron size sphere was affixed to the end of the cantilever. Because the ball was much larger than the amplitude observed in the experiment, the tip-sample distance could be considered as a fixed value. The squeeze film force was considered as concentrating on the sphere [25]. For the convenience of the theoretical calculation, the squeeze film damping between the ball probe and the sample was simplified to a one-dimensional oscillator model.

The tip-sample action force can be written as:

(1) $$\begin{array}{c} F=6\mu \pi R^{2}V/z, \end{array}$$

where *μ* = 1.79 × 10^−5^Pa · s is the viscosity of the air, *R* is the radius of the sphere (~10 um), *V* is the velocity of the sphere, and *z* is the sphere sample distance [25].

The distribution of the squeeze film force mainly depends on the instantaneous velocity of the sphere and the distance *z*. The amplitude of the vibration system is near 50 nm, and the frequency is 200 kHz. In the ball probe experiment, the truncation did not appear, and the small ball did not touch the sample. The squeeze film force reduced the amplitude until the system could no longer present a stable image.

The one-dimensional damping system can be expressed as follows:

(2) $$\begin{array}{c} m\ddot{x}+c\dot{x}+kx=f\left(t\right). \end{array}$$

In Eq. (2), *m*, *c*, *k*, and *f*(*t*) are the equivalent mass, equivalent damping coefficient, equivalent stiffness, and equivalent excitation of the cantilever, respectively. Especially, for a cantilever beam with a concentrated mass ball at the free end, *m* = *m*_*b*_ + 0.24*m*_*l*_, $k=\sqrt{3EI/L^{3}}$, where *m*_*b*_ is the mass of the microball, *m*_*l*_ indicates the mass of the cantilever, *E* represents the elastic modulus, *I* indicates the cross-sectional moment of inertia, and *L* is the cantilever length. Linear damping consists of squeeze film damping and constant damping (air damping and internal damping). The squeeze film damping is *c*_*sq*_ = 6*πμR*^2^/*z*, and the constant damping is *c*_*con*_; then, *c* = *c*_*sq*_ + *c*_*con*_[22]. The system quality factor *Q* can be obtained from *c*.

(3) $$\begin{array}{c} Q=\sqrt{km}/\left(6\pi \mu R^{2}/z+c_{con}\right) \end{array}$$

**Figure 3:**
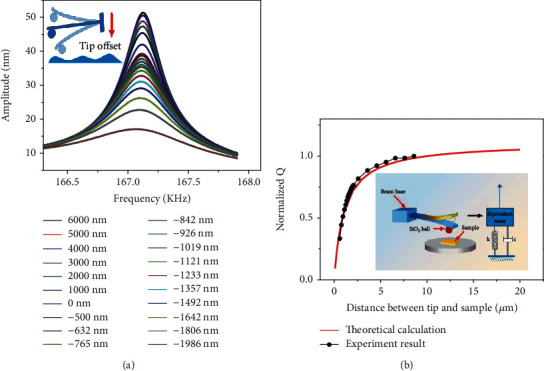
(a) Ball probe experimental sweep curves for different tip offset (relative tip-sample distances). (b) Comparison of the experimental results and the theoretical calculations of the ball probe.

Eq. (3) can be used to calculate the theoretical quality factor *Q* of the ball probe with microspheres and is a function of the tip-sample distance *z*. The tuning experiment results are shown in Figure 3(a), the largest amplitude of each resonance curve was also recorded, the theoretical calculations were completed using Eq. (3), and the experimental results were obtained by experimental sweep curves. For different tip-sample distances, the experimental quality factors and the theoretical curves calculated by Eq. (3) were normalized, shown in Figure 3(b). In the ball probe tuning experiment, the tip-sample distance was approximately in the micron range and van der Waals forces could be ignored in this condition. It was clearly seen that the normalized experimental *Q* variation trend was consistent with the theoretical calculation results. The one-dimensional oscillator model is reasonable for the ball probe tuning experiment.

### 3.3. Tipless Probes

For a small cantilever sample distance, the squeeze film damping between the cantilever and sample can be expressed by the Reynolds equation [16, 24]:

(4) $$\begin{array}{c} \frac{\partial }{\partial x}\left(z^{3}\frac{\partial p}{\partial x}\right)+\frac{\partial }{\partial y}\left(z^{3}\frac{\partial p}{\partial y}\right)=12\mu \frac{\partial z}{\partial t}, \end{array}$$

where *p* is the pressure in the film, *x* is the axis parallel to the beam length, and *y* is the axis parallel to the beamwidth.

Combining with the actual size of the cantilever, the width-length ratio is *X* = *b*/*L* = 0.2, where *b* is the cantilever width. According to Pandey [23], the Reynolds equation can be simplified to one dimension and the errors can be neglected. As the curvature radius along *x* direction being less than *y* direction, the Reynolds equation can be simplified to

(5) $$\begin{array}{c} \frac{\partial }{\partial }\left(z^{3}\frac{\partial p}{\partial y}\right)=12\mu \frac{\partial z}{\partial t}. \end{array}$$

The pressure *p* is obtained by integrating by Eq. (5) with the boundary condition *p* = 0 at *y* = ±*b*/2. As the cantilever sample distance is much larger than the vibration amplitude *w*, the squeeze force per unit length can be simplified to

(6) $$\begin{array}{c} F_{y}=\int_{-b/2}^{b/2}-pdy=\frac{\mu b^{3}}{z^{3}}\frac{\partial w}{\partial t}. \end{array}$$

The equation of motion for the microcantilever with damping (consisting of squeeze film damping and constant damping) can be written as [24]:

(7) $$\begin{array}{c} \rho bh\frac{\partial^{2}w}{\partial t^{2}}+\left(\frac{\mu b^{3}}{z^{3}}+c^{\prime }\right)\frac{\partial w}{\partial t}+EI\frac{\partial^{4}w}{\partial x^{4}}=f\exp\left(i\omega t\right), \end{array}$$

where *ρ* indicates the volume density of the cantilever, *h* indicates the cantilever thickness, *c*′ represents the constant damping coefficient per unit length. For a inclined cantilever with an angle *α* ~ 10°, the cantilever-sample distance *z* can be expressed as *z* = *d* + (*L* − *x*)sin*α*, where *d* is the distance between the free end of the cantilever beam and the sample at equilibrium. By using modal decomposition, i.e., *w*_*i*_(*x*, *t*) = ∑_*i*=1_^∞^*φ*_*i*_(*x*)*q*_*i*_(*t*), Eq. (7) can be expressed as following when only first-order is considered.

(8) $$\begin{array}{c} \begin{array}{c} {\ddot{q}}_{1}\left(t\right)+{\left(\psi_{1}\rho bh\right)}^{-1}\int \left(\frac{\mu b^{3}}{z^{3}}+c^{\prime }\right){\varphi_{1}}^{2}dx{\dot{q}}_{1}\left(t\right)+\frac{EI}{\rho bh}k_{1}^{4}q_{1}\left(t\right)\\ ={\left(\psi_{1}\rho bh\right)}^{-1}F_{exc}\left(t\right)\int \varphi_{1}\left(x\right)dx, \end{array} \end{array}$$

where *ψ*_1_ = ∫*φ*_1_^2^*dx*, $k_{1}=\sqrt[{4}]{\rho bd\omega_{1}^{2}/EI}$. Therefore, the system damping ratio can be expressed as *ζ*_*sum*_ = (2*ψ*_1_*ρbdω*_1_)^−1^∫*μb*^3^/*z*^3^*φ*_1_^2^*dx* + *ζ*_*con*_, where *ω*_1_ is the first-order resonance frequency, and *ζ*_*con*_ indicates the constant damping ratio. The system *Q* can then be obtained by *ζ*_*sum*_.

(9) $$\begin{array}{c} Q=1/2\zeta_{sum}. \end{array}$$

**Figure 4:**
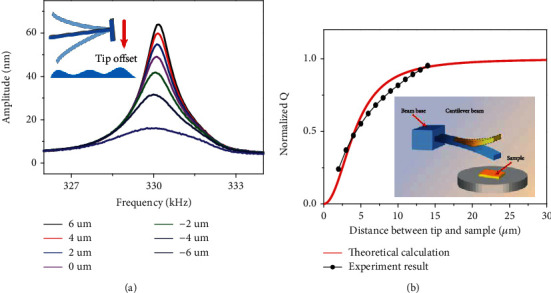
(a) Tipless probe experimental sweep curves for different tip offset (relative tip-sample distances). (b) Comparison of the experimental results and the theoretical calculations of the tipless probe.

Eq. (9) can be used to calculate the theoretical quality factor *Q* of tipless probe; the value of *ζ*_*con*_ is usually ~0.001. The tuning experiment results are shown in Figure 4(a), the theoretical calculations were completed using Eq. (9), and the experimental results were obtained by experimental sweep curves, together with the largest amplitude in each curve. The normalized experimental and theoretical quality factors for different tip-sample distances are also shown in Figure 4(b). It is clear that the trend of the normalized experimental quality factor is consistent with the theoretical results. The simplified model is in good agreement with the tipless probes in the tuning experiment.

**Figure 5:**
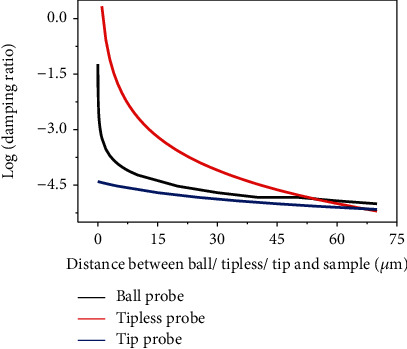
Theoretical squeeze film effect region for different probe in different distance.

For the three types of probes, the theoretical calculation squeeze film damping ratio is shown in Figure 5. Clearly, the squeeze film damping has different effect region on different probes. At the same tip-sample distance, the influence of squeeze film damping on different probes is also very different.

## 4. Conclusions

In summary, the effect of the squeeze film force is a nonnegligible factor for the damping when the tip-sample distance is in the range of several micrometers, especially in TM-AFM. The experimental results and theoretical model data were in good agreement, and the simplified models are credible and easy to calculate. For the ball probe, the squeeze film force was mainly concentrated on the sphere which was attached to the end of the cantilever. For tipless probes, the main part of the squeeze film force was concentrated between the cantilever and the sample, distributed over the entire cantilever beam. For tip probes, long and thin tips could increase the distance between the cantilever and sample, and high distances reduced the squeeze film force when the tip-sample distance was in the nanometer range. This study is of great significance for understanding the mechanism of squeeze film damping dissipation in TM-AFM and promoting the development of AFM.

## Data Availability

The data used to support the findings of this study are available from the corresponding author upon request.

## References

[1] Binnig G., Quate C. F., Gerber C. (1986). Atomic Force Microscope. *Physical Review Letters*.

[2] Trache A., Meininger G. A. (2008). Atomic force microscopy (AFM). *Current Protocols in Microbiology*.

[3] Stark R. W., Drobek T., Heckl W. M. (1999). Tapping-mode atomic force microscopy and phase-imaging in higher eigenmodes. *Applied Physics Letters*.

[4] Tamayo J., Garcia R. (1998). Relationship between phase shift and energy dissipation in tapping-mode scanning force microscopy. *Applied Physics Letters*.

[5] Cleveland J. P., Anczykowski B., Schmid A. E., Elings V. B. (1998). Energy dissipation in tapping-mode atomic force microscopy. *Applied Physics Letters*.

[6] Tamayo J. (1999). Energy dissipation in tapping-mode scanning force microscopy with low quality factors. *Applied Physics Letters*.

[7] Zitzler L., Herminghaus S., Mugele F. (2002). Capillary forces in tapping mode atomic force microscopy. *Physical Review B*.

[8] Duque J. S., Gutierrez A., Cortés D. (2020). Dynamics of a micro-electro-mechanical system associated with an atomic force microscope considering squeeze film damping. *Applied Optics*.

[9] Sahagún E., García-Mochales P., Sacha G. M., Sáenz J. J. (2007). Energy dissipation due to capillary interactions: hydrophobicity maps in force microscopy. *Physical Review Letters*.

[10] Santos S., Thomson N. H. (2011). Energy dissipation in a dynamic nanoscale contact. *Applied Physics Letters*.

[11] Garcia R., Gómez C. J., Martinez N. F., Patil S., Dietz C., Magerle R. (2006). Identification of nanoscale dissipation processes by dynamic atomic force microscopy. *Physical Review Letters*.

[12] Haider S. T., Saleem M. M., Ahmed M. (2019). Effect of environmental conditions and geometric parameters on the squeeze film damping in RF-MEMS switches. *Analog Integrated Circuits and Signal Processing*.

[13] Wei Z., Sun Y., Ding W. X., Wang Z. R. (2016). The formation of liquid bridge in different operating modes of AFM. *Science China Physics, Mechanics & Astronomy*.

[14] Chen L., Yu X., Wang D. (2007). Cantilever dynamics and quality factor control in AC mode AFM height measurements. *Ultramicroscopy*.

[15] Fairbairn M., Moheimani S. O. R. (2013). Sensorless enhancement of an atomic force microscope micro-cantilever quality factor using piezoelectric shunt control. *Review of Scientific Instruments*.

[16] Bao M., Yang H. (2007). Squeeze film air damping in MEMS. *Sensors and Actuators A Physical*.

[17] Paulo A. S., Garcia R. (2001). Tip-surface forces, amplitude, and energy dissipation in amplitude-modulation (tapping mode) force microscopy. *Physical Review B*.

[18] Garcia R., Perez R. (2002). Dynamic atomic force microscopy methods. *Surface Science Reports*.

[19] Hoummady M., Farnault E. (1998). Enhanced sensitivity to force gradients by using higher flexural modes of the atomic force microscope cantilever. *Applied Physics A: Materials Science & Processing*.

[20] Zhao Y., Huang Q., Zhang L., Zhang Y., Cheng R. (2017). Squeeze film air damping in tapping mode atomic force microscopy. *Micromachines*.

[21] Abtahi M., Vossoughi G., Meghdari A. (2014). Effects of the van der Waals force, squeeze-film damping, and contact bounce on the dynamics of electrostatic microcantilevers before and after pull-in. *Nonlinear Dynamics*.

[22] Harrison C., Tavernier E., Vancauwenberghe O. (2007). On the response of a resonating plate in a liquid near a solid wall. *Sensors and Actuators A Physical*.

[23] Pandey A. K., Pratap R. (2007). Effect of flexural modes on squeeze film damping in MEMS cantilever resonators. *Journal of Micromechanics and Microengineering*.

[24] Hosaka H., Itao K., Kuroda S. (1995). Damping characteristics of beam-shaped micro-oscillators. *Sensors and Actuators A Physical*.

[25] Lévêque G., Girard P., Belaidi S., Cohen Solal G. (1997). Effects of air damping in noncontact resonant force microscopy. *Review of Scientific Instruments*.

